# Deciphering Paroxysmal Nocturnal Hemoglobinuria: An Unusual Paradigm of Hemolytic Anemia

**DOI:** 10.7759/cureus.67194

**Published:** 2024-08-19

**Authors:** Soumya Athota, Sowmya Gopalan, Preetam Arthur, Ananthvikas Jayaram, Anjali Satish

**Affiliations:** 1 Internal Medicine, Sri Ramachandra Institute of Higher Education and Research, Chennai, IND; 2 Hematopathology, Anand Diagnostic Laboratory, Bengaluru, IND

**Keywords:** drug-induced immune hemolytic anemia, pnh flow cytometry, eculizumab, aretrial and venous thromboses, sub clinical clones, pnh

## Abstract

Paroxysmal nocturnal hemoglobinuria (PNH), a clonal hematopoietic stem cell disorder, arises from the increased sensitivity of red blood cells (RBC) to complement due to an acquired deficiency of certain glycosylphosphatidylinositol (GPI)-linked proteins, resulting in chronic intravascular hemolysis, arterial and venous thrombotic phenomena, multi-organ damage, and failure. We present an intriguing case of hemolytic anemia, initially suspected to be drug-induced, and later found to be associated with PNH, despite being a subclinical clone. A clinician should not hesitate to repeat fluorescent-labeled aerolysin (FLAER) cytometry if the clinical picture strongly favors a diagnosis of PNH. This case marks the importance of testing for PNH clones in autoimmune hemolytic anemia (AIHA) since their prevalence is not negligible and may correspond to a prominent hemolytic pattern, a higher thrombotic risk, and a higher therapeutic indication, such as eculizumab. This underscores the significance of conducting a thorough evaluation for occult causes of treatment-unresponsive hemolytic anemia, paving options for an early and alternative therapeutic approach.

## Introduction

As a result of a somatic mutation in the PIGA (phosphatidylinositol glycan class A) gene on chromosome X, people with paroxysmal nocturnal hemoglobinuria (PNH) lack a certain protein. This enzyme is necessary for the synthesis of a glycosylphosphatidylinositol (GPI) anchor, which many cell membrane proteins bind to. A partial or complete absence of enzyme leads to complement-mediated red cell lysis [1]. Based on the clone size, which can be small or large, PNH can cause a wide range of symptoms, such as anemia, shortness of breath, abdominal pain, thrombosis, damage to end organs, and bone marrow failure (BMF). One of the most common causes of mortality and morbidity is thromboembolism; other causes include acute myeloid leukemia (AML), myelodysplastic syndrome (MDS) or aplastic anemia (AA) associations, hemorrhage, renal and cardiac failure, and infections [2]. Flow cytometry (FCM) is currently the gold standard diagnostic modality for the detection of GPI-AP-deficient RBCs and leucocytes, the marker being fluorescent-labeled aerolysin (FLAER). Eculizumab, a complement inhibitor is the approved drug for PNH currently [3].

## Case presentation

A female in her 60s with no prior co-morbidities presented with complaints of straightforward fatiguability, bilateral lower limb swelling, and shortness of breath for 15 days. There was no history of oliguria or chest pain, but significant weight loss was notable. On examination, there were signs of frank failure. Hemoglobin (Hb) levels in the lab were 5.2, and other cell types were normal. Peripheral smear showed macrocytic anemia. Liver function test (LFT), reticulocyte count, iron studies, RBC folate, and vitamin B12 levels were within normal limits (Table 1).

**Table 1:** Laboratory findings with normal reference values WBC: white blood cells; RBC: red blood cells; MCV: mean corpuscular volume; LDH: lactate dehydrogenase; TIBC: total iron binding capacity; G6PD: glucose 6 phosphate dehydrogenase; ACE: angiotensin-converting enzyme

Investigation	Patient Value	Reference Range
Hemoglobin	5.1	12-15 gm/dL
Total Counts (WBC)	6500	4000-11000 cells/mm^3^
RBC	3.1	3.8-4.8 million/mm^3^
Platelets	3.5	1.5-4.5 lakhs/mm^3^
MCV	106	83-101 FL
LDH	1707	208-378 U/L
Serum Iron	54	60-180 ug/dL
TIBC	269	240-250 ug/dL
Serum Ferritin	191	13-150 ng/mL
Reticulocyte Count	14.7	0.5-2.5%
Serum Vitamin B12	404	197-771 pg/mL
RBC Folic Acid	19.8	4.6-34.8 ng/mL
Total Bilirubin	3.49	0.3-1.2 mg/dL
Indirect Bilirubin	2.71	0.1-1.0 mg/dL
G6PD Levels (Quantitative)	31.9	6.4-18.8 U/g Hb
Serum ACE	48	13.3-63.9 U/L

**Figure 1:**
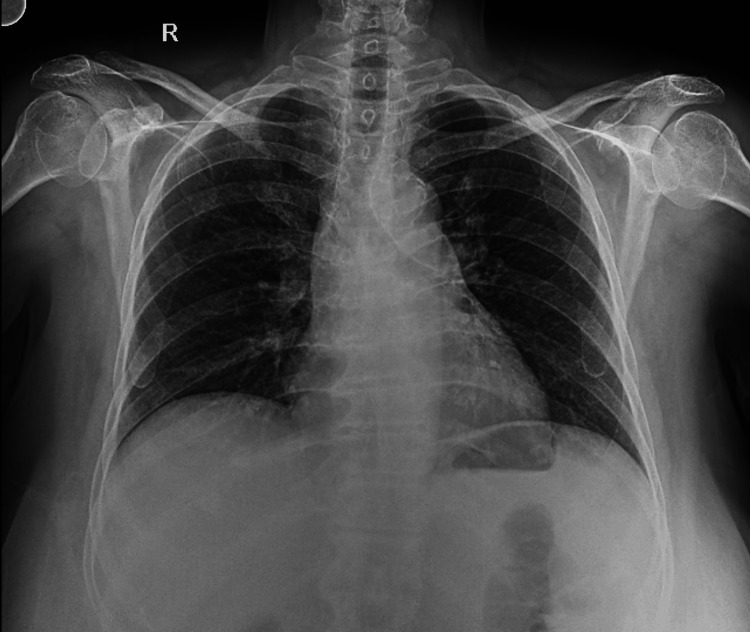
Radiograph of the chest

**Figure 2:**
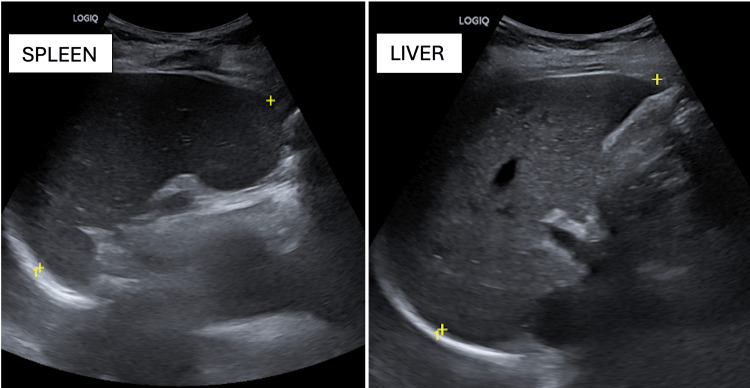
An ultrasonogram of the abdomen without hepatosplenomegaly

**Figure 3:**
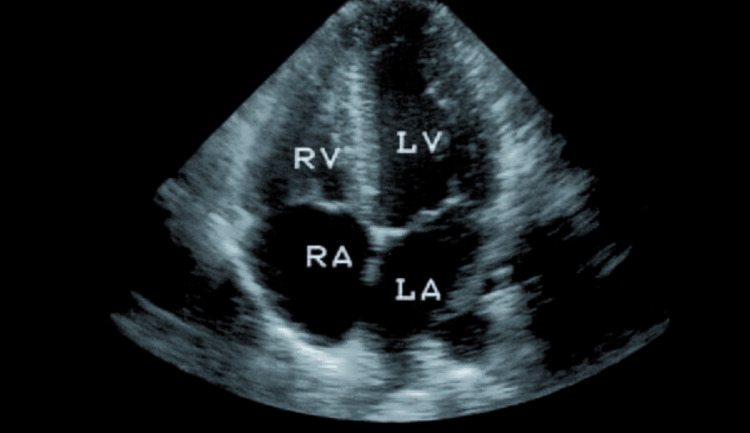
A normal echocardiogram (apical four-chamber view), without any left ventricular dysfunction or regional wall motion abnormality RA: right atria; LA: left atria; RV: right ventricle; LV: left ventricle

With a normal echocardiogram and normal pulmonary artery pressures, an ultrasound abdomen showed no organomegaly. X-ray of the chest was normal (Figures 1-3).

**Figure 4:**
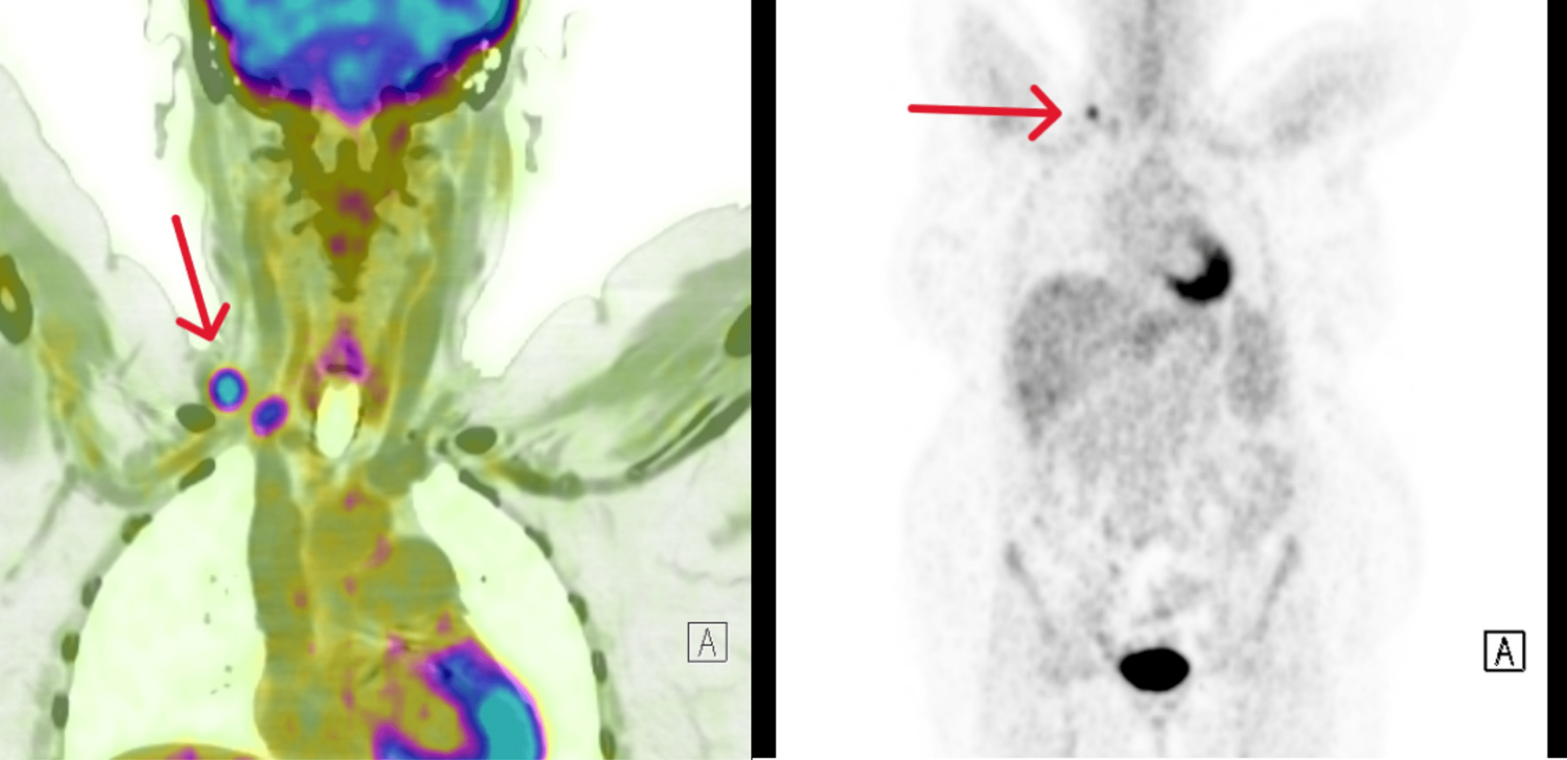
PET-CT scan of the whole body showing FDG uptake (SUV 11.23) in an isolated right supraclavicular lymph node (12 x 9 mm), with no significant uptake observed elsewhere. PET-CT: positron emission tomography-computed tomography; FDG: fluorodeoxyglucose; SUV: standardized uptake value

We transfused her with two packs of cells. With a background of weight loss and anemia in failure, and considering a possible occult malignancy, we proceeded with a positron emission tomography-computed tomography (PET-CT) of the whole body, which picked up an isolated right supraclavicular lymphadenopathy with no uptake elsewhere (Figure 4).

**Figure 5:**
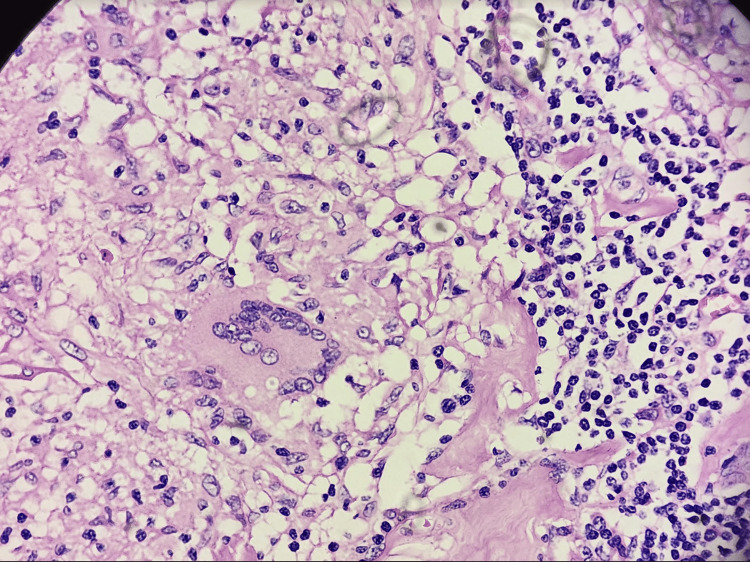
Microphotograph showing multiple epithelioid granulomas with multinucleated giant cells in a background of lymphocytes (H&E, 100X).

We performed an excisional biopsy, and the histology confirmed the presence of granulomatous lymphadenitis as portrayed in Figure 5.

Chest X-ray and serum angiotensin convertase enzyme (ACE) levels were normal, and the Mantoux test was negative. We empirically started her on anti-tuberculosis therapy (ATT) and discharged her with a Hb level of 8 g/dL. Two weeks later, she presented with a decreased appetite and yellowish discoloration of the sclera. There was no history of dark-colored or blood-stained urine. Further tests, as tabulated in Table 2, showed that the Hb level had dropped to 4.1, along with indirect hyperbilirubinemia. A hemolysis workup revealed elevated reticulocyte count and lactate dehydrogenase (LDH) levels, and a peripheral smear revealed fragmented RBCs. To rule out glucose 6 phosphate dehydrogenase deficiency, the G6PD (quantitative) level was done, which was normal 31.9 U/gm Hb (ref: 6.4-18.8), the haptoglobin level was <0.0781 (ref: 0.2-3.0 gm/L), and the cold agglutination test came back negative. The anti-nuclear antibodies tested by IF were negative. Her direct Coombs test (DCT) was negative, whereas her indirect Coombs test (ICT) showed a weakly positive result (probable transfusion-related). We biopsied the bone marrow to rule out MDS or other causes of BMF and found erythroid hyperplasia, hypercellular marrow, and normal myeloid maturation. Having ruled out other possible etiologies, suspecting rifampicin as the cause of hemolysis, we modified ATT and initiated steroids. She has become transfusion-dependent.

**Table 2:** Investigations with respect to the timeline of disease progression ATT: anti-tuberculosis therapy

Timeline	At first visit	After ATT initiation	On steroids	Review, despite steroids	Normal range
Hemoglobin (gm/dL)	5.2	4.1	6.2	5.8	12-15 gm/dL
Total leucocytes (cells/mm^3^)	6448	9864	8788	7640	4000-11,000 cells/mm^3^
Platelets (lakh cells/mm^3^)	3.84	4.02	3.61	5.66	1.5-4.5 lakhs /mm^3^
Lactate dehydrogenase (U/L)	284	1645	478	892	208-378 U/L
Peripheral smear	Macrocytic anemia	Fragmented red cells	Dimorphic picture	Dimorphic picture	Normal study

**Figure 6:**
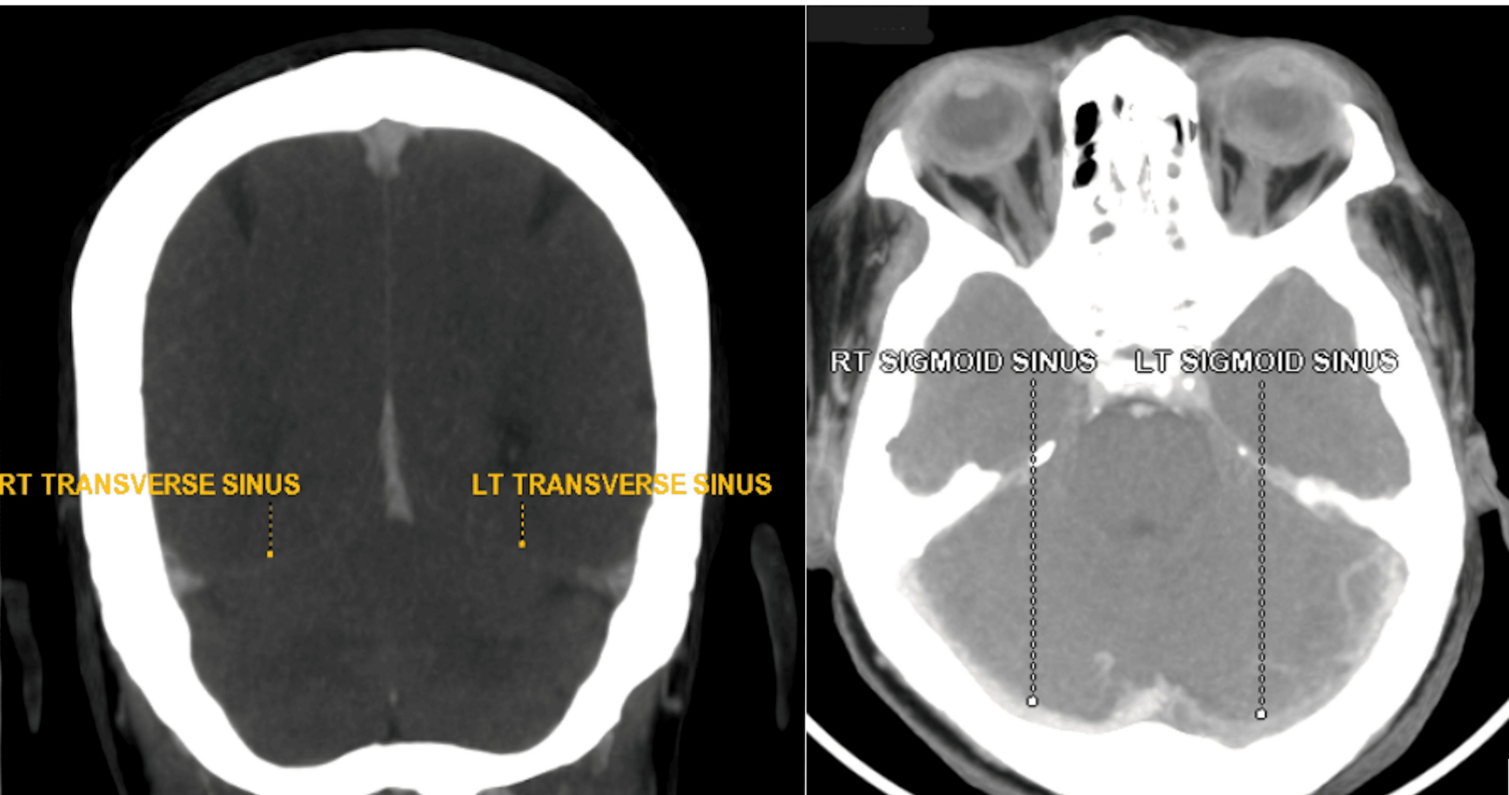
CT cerebral venogram showing partial thrombosis in the left sigmoid sinus and left transverse sinus.

Despite adherence to steroids, two months later she presented with a new-onset severe headache and persistently low Hb. DCT and ICT were both negative this time. In both eyes, the fundus showed fulminant papilledema. A CT venogram and MR brain imaging showed that the left sigmoid sinus, transverse and straight sinuses, and proximal bilateral internal jugular veins were all thrombosed (Figure 6).

**Figure 7:**
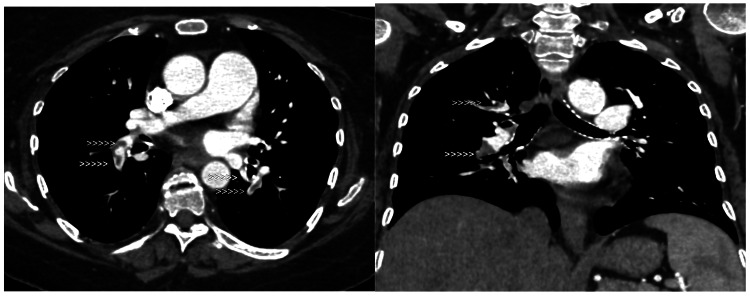
CT pulmonary angiogram showing acute pulmonary thromboembolism.

We initially started the patient on low-molecular-weight heparin (LMWH) and later switched to apixaban. We continued the steroids and added azathioprine, assuming the patient had DCT-negative hemolytic anemia. Concomitant hemolysis and thrombotic phenomena raised suspicions of PNH, prompting the use of FLAER cytometry, which did not detect a PNH clone. Two months later, she presented with persisting hemolysis and lower limb cellulitis. The dopplers of the bilateral lower limbs showed deep vein thrombosis. We continued the anticoagulation and administered antibiotics. For an episode of acute-onset shortness of breath with desaturation, a CT pulmonary angiogram (CTPA) revealed acute pulmonary thromboembolism with no evidence of pulmonary infarction (Figure 7).

**Figure 8:**
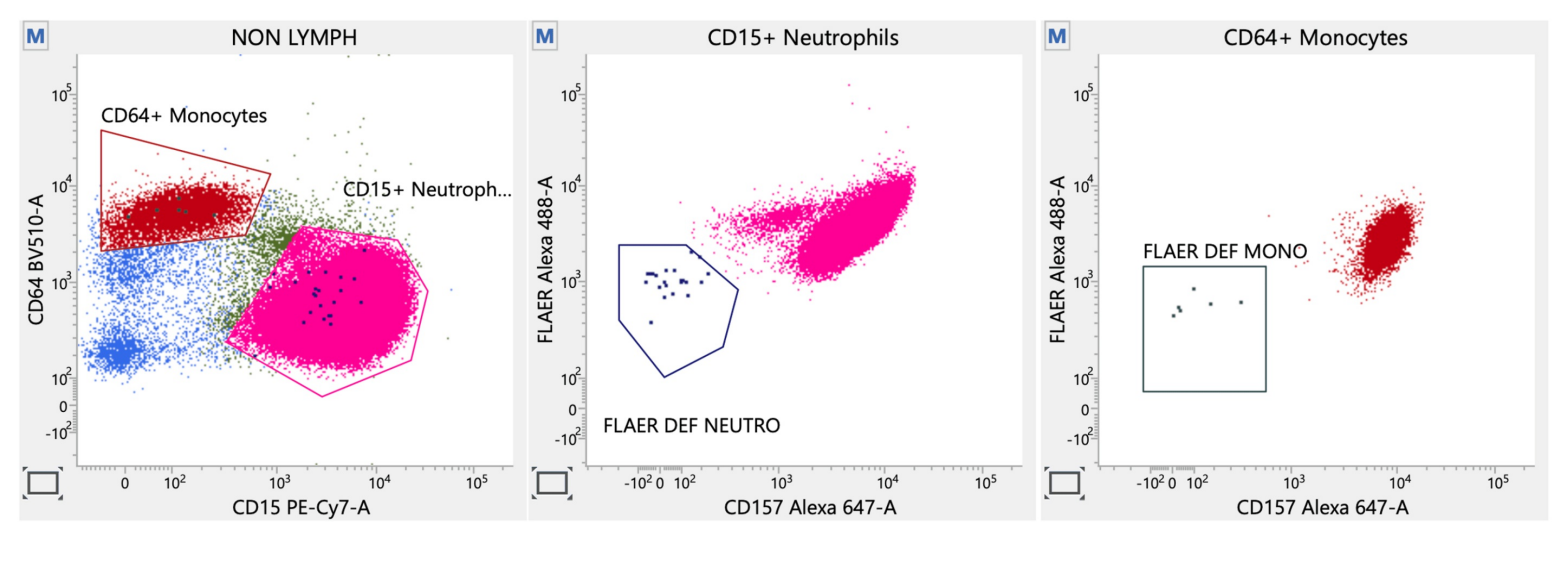
Flow cytometry analysis for fluorescent-labeled aerolysin (FLAER)-deficient neutrophils and monocytes, which were identified using specific non-glycosylphosphatidylinositol (GPI) dependent markers CD15 and CD64, respectively (left corner); The center and right panels show a combination of FLAER and CD157 used to identify CD157-negative neutrophils and monocytes.

In view of recurrent venous thrombosis, the antiphospholipid antibody (APLA) profile was done, came out to be negative, and thrombophilia panel markers were within normal limits. Given the high level of clinical suspicion, we repeated a highly sensitive FCM PNH assay with CD157, which revealed a small PNH clone containing 0.01% neutrophils and 0.08% monocytes as charted in Figure 8.

**Figure 9:**
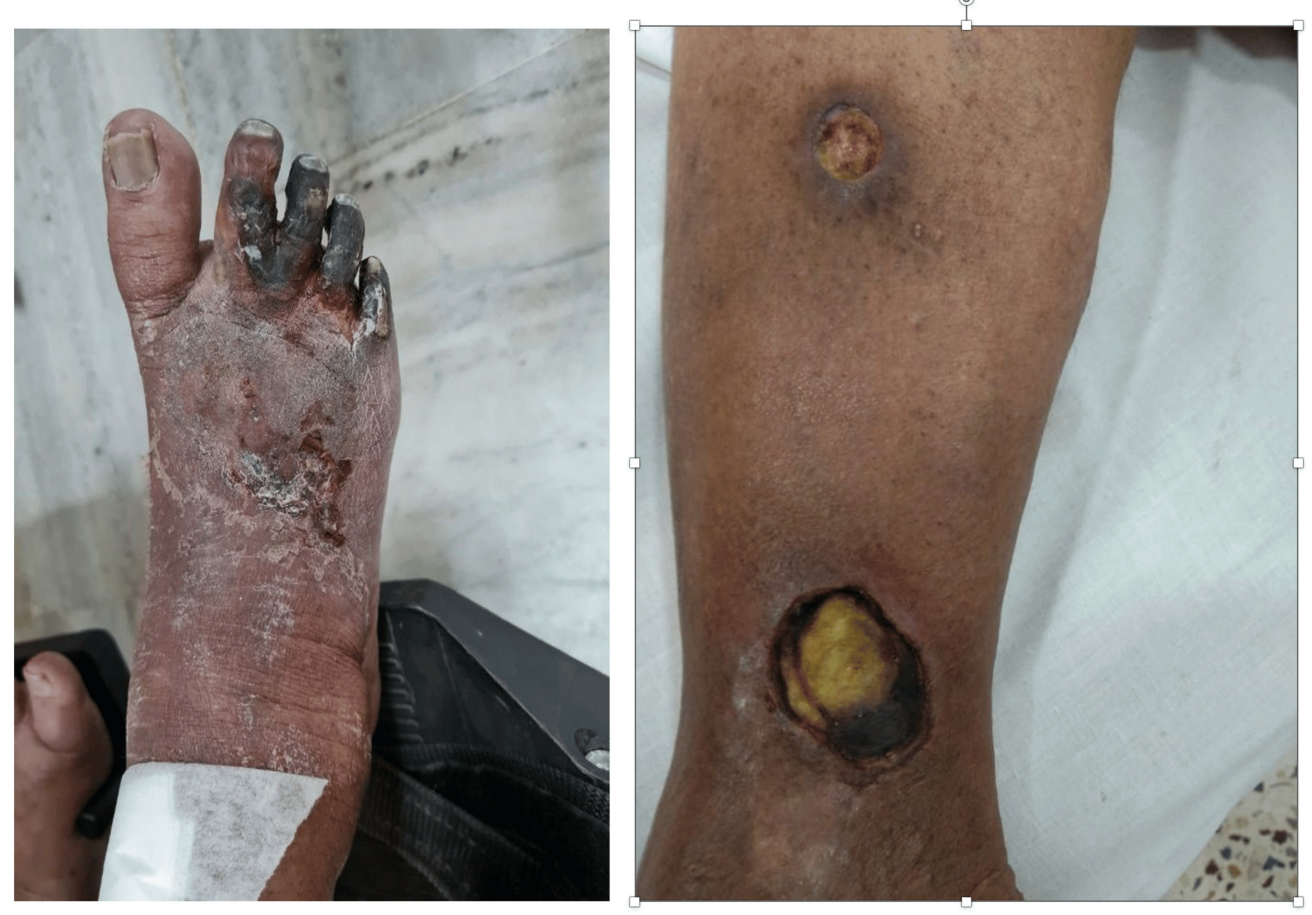
Pre-gangrenous changes with arterial ulcers in the lower limb.

**Figure 10:**
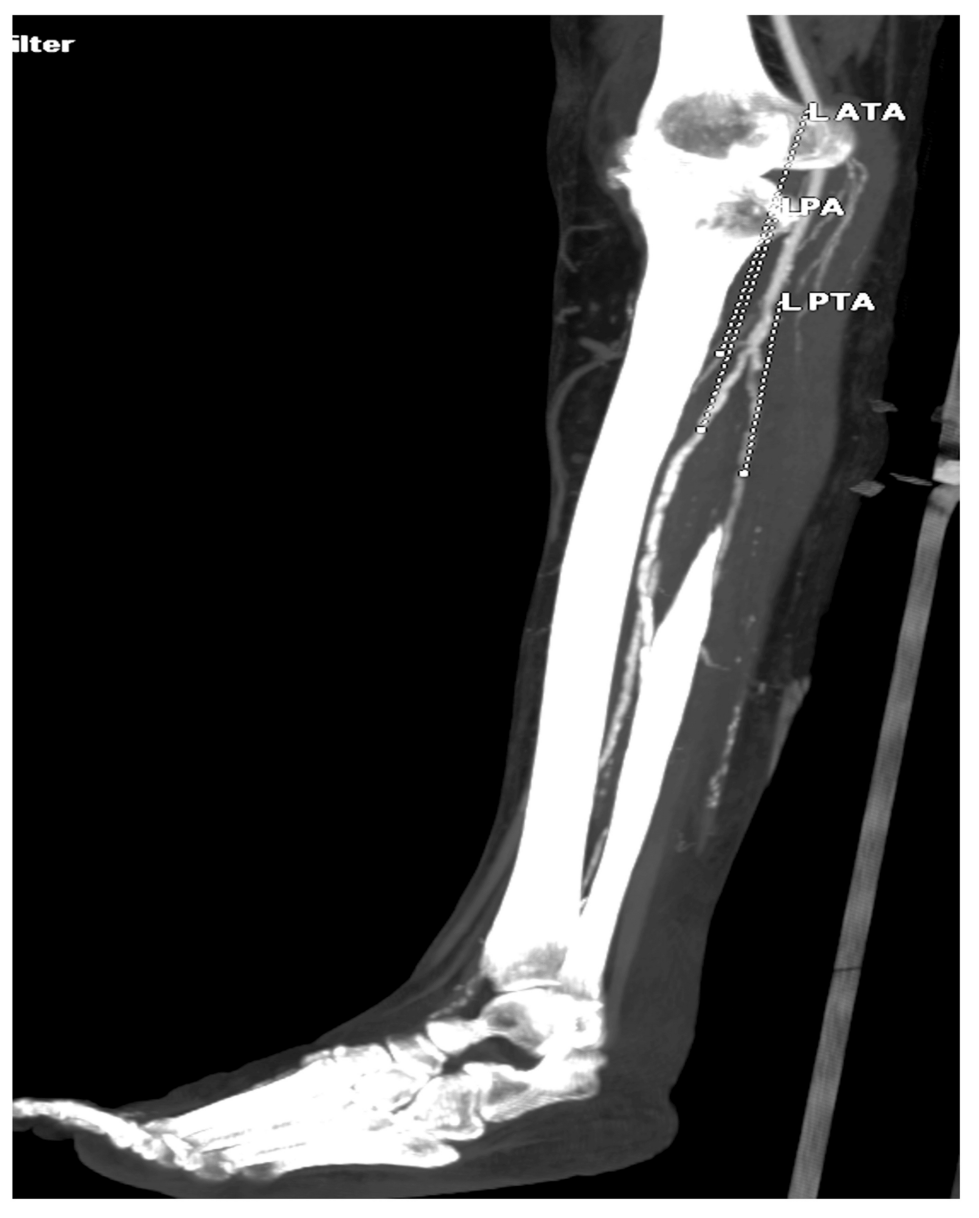
Features of peripheral arterial disease in a CT arteriogram of the lower limb vessels.

We started her on danazol, an androgenic hormone. After a month, she developed arterial ulcers in the calf with necrotic base and superadded infection, with feeble peripheral pulses and pre-gangrenous changes in the right foot as shown in Figure 9. CT arteriography revealed peripheral arterial disease involving the bilateral posterior tibial artery, dorsalis pedis artery, and right peroneal artery (Figure 10).

Vascular surgery intervened and performed tibial angioplasty. The edge biopsy of the wound was consistent with vasculitis; however, the systemic vasculitis workup antibodies perinuclear antineutrophil cytoplasmic antibodies (P-ANCA) and cytoplasmic antineutrophil cytoplasmic antibodies (C-ANCA), as well as the line immune assay, were all negative. Gangrene worsened, leading to a right forefoot amputation. She developed a *Clostridium difficile *infection treated with oral vancomycin. With a failing heart, acute kidney injury worsening acidosis, underlying hemolysis Hb 7.7, and ongoing gram-negative septicemia, she succumbed over the course to multiorgan dysfunction syndrome (MODS).

## Discussion

Conditions apart from PNH that can cause both arterial and venous thromboses are anti-phospholipid antibody syndrome (ALPS), myeloproliferative neoplasms, heparin-induced thrombocytopenia (HIT), microangiopathic hemolytic anemia (MAHA), and disseminated intravascular coagulation (DIC) [1]. Ours was a case of DCT-negative hemolytic anemia with a positive PNH clone, thrombotic phenomena such as bilateral lower limb venous thrombosis, pulmonary thromboembolism, cerebral venous thrombosis, and peripheral arterial disease with arterial ulcers. Considering that this patient was on ATT, there is a higher possibility of rifampicin-induced hemolysis being the cause. Despite rifampicin discontinuation, a further drop in Hb and thromboses, both arterial and venous led to screen for other causes of hemolysis, establishing an association with subclinical PNH clones. Aside from transfusion dependence, we treated her with apixaban, a single antiplatelet, along with statin, iron, and folate supplements. Though there are no proven clinical trials, our patient showed some clinical benefit from the combination of corticosteroid and danazol. Persistent hemolysis can be destructive with life-threatening consequences, devastating to the family; thrombosis followed by infections is the leading cause of mortality.

Three main categories primarily classify PNH: classic, subclinical, and in the context of another specific bone marrow disorder. The RBCs that come from PNH clones don't have enough of the surface membrane complement regulatory proteins CD55 and CD59. This makes them very sensitive to activated complement lysis. Blocking complement with the humanized anti-C5 monoclonal antibody eculizumab is very good at reducing intravascular hemolysis, lowering the risk of thrombosis, and lowering the need for transfusions, all of which improve quality of life. Despite being the preferred drug for PNH management, the financial burden of long-term administration of eculizumab necessitates lifelong anticoagulation as a secondary thrombotic prophylaxis [2]. The Modified Ham's test, the sucrose lysis test, and the gel card technique (GCT) are now obsolete tests for a diagnosis of PNH. A single-tube assay combining FLAER with a GPI-linked protein on neutrophils or monocytes (CD157, CD24, CD14, etc.) is now the recommended standard of care with a high degree of sensitivity, precision, and reduced cost of screening, thus replacing the CD55 and CD59-based RBC assays available earlier [3].

The DCT, which is known to be negative in 3-11% of autoimmune hemolytic anemia (AIHA) cases, can detect the immune nature of AIHA. Other laboratory investigations, such as persistently dropping Hb, hyperbilirubinemia, low haptoglobin, and high LDH, and reticulocyte count, emphasize the presence of DCT-negative hemolytic anemia [4,5]. Despite their sensitivity, FCM assays can adversely impact leukopenia samples due to their detection limit [6]. PNH and AIHA occasionally occur concomitantly. The inflammatory/thrombotic process in PNH may cause a conformational change on the erythrocyte surface, which triggers an immune antibody response; alternatively, autoantibodies in AIHA may facilitate clonal expansion of the GPI-deficient cell [7]. Clinical and serological findings alone cannot verify a true diagnosis of PNH or AIHA, a comprehensive analysis of laboratory and clinical data is essential for optimal therapy. Immunosuppression is the mainstay of treatment; in cases of steroid failure, rituximab may be considered.

Studies have shown that early initiation of specific immunosuppression therapy in PNH positivity, at any clone size, has led to a dramatic reduction in the incidence of mortality even for a clone size of 0.01% [7]. Thus, a clinician should not hesitate to repeat FLAER cytometry if the clinical picture strongly favors a diagnosis of PNH. PNH clones, which are associated with thrombotic events proportional to the size of the clone, increase the risk of immune-mediated BMF in patients with AA (40-70% of cases) and MDS (12%-17.6% of cases) [8,9]. In our case, we detected the PNH clone for the second time, despite its subclinical nature, without classifying it as either classical or hypoplastic. This case shows how important it is to check for PNH clones in AIHA because they are common and may be linked to a clear pattern of hemolysis, a higher risk of thrombosis, and a stronger therapeutic indication, like eculizumab. Subclinical PNH requires no specific therapy, with attention given to BMF syndrome. For PNH/BMF syndrome, the focus is on treating BMF, and patients with significant PNH clones may benefit from eculizumab. Classic PNH is treated with eculizumab [10].

## Conclusions

PNH should be considered when Coombs-negative hemolysis occurs concomitantly with arterial and venous thromboses, even without obvious hemoglobinuria. PNH clone testing is advisable in complex cases with an inadequate response to AIHA-specific therapy. A true diagnosis of AIHA or PNH can neither be verified by clinical nor serological findings alone; a collective picture of both remains obligatory for fulfilling the criteria of optimal diagnosis and therapy. Persistent hemolysis can be destructive, and the consequences may be life-threatening and devastating to the family, with thrombosis followed by infections being the leading cause of mortality. Though consensus states eculizumab as the treatment of choice in classical PNH and as a probable benefit in marrow failure association syndromes, there is no clear data on its role in AIHA with small PNH clones causing significant clinical disease patterns, thus paving the way for further studies.
